# Complete sequence of kenaf (*Hibiscus cannabinus*) mitochondrial genome and comparative analysis with the mitochondrial genomes of other plants

**DOI:** 10.1038/s41598-018-30297-w

**Published:** 2018-08-24

**Authors:** Xiaofang Liao, Yanhong Zhao, Xiangjun Kong, Aziz Khan, Bujin Zhou, Dongmei Liu, Muhammad Haneef Kashif, Peng Chen, Hong Wang, Ruiyang Zhou

**Affiliations:** 1 College of Life Sciences and Technology, Guangxi University, Nanning, 530005 China; 2 Key Laboratory of Plant Genetic and Breeding, College of Agriculture, Guangxi University, Nanning, 530005 China; 3 Cash Crop Institute of Guangxi Academy of Agricultural Sciences, Nanning, 530007 China; 4 Key Laboratory of Plant-Microbe Interactions, Department of Life Science and Food, Shangqiu Normal University, Shangqiu, 476000 China; 5 Department of Biochemistry, University of Saskatchewan, Saskatoon, SK S7N5E5 Canada

## Abstract

Plant mitochondrial (mt) genomes are species specific due to the vast of foreign DNA migration and frequent recombination of repeated sequences. Sequencing of the mt genome of kenaf (*Hibiscus cannabinus*) is essential for elucidating its evolutionary characteristics. In the present study, single-molecule real-time sequencing technology (SMRT) was used to sequence the complete mt genome of kenaf. Results showed that the complete kenaf mt genome was 569,915 bp long and consisted of 62 genes, including 36 protein-coding, 3 rRNA and 23 tRNA genes. Twenty-five introns were found among nine of the 36 protein-coding genes, and five introns were *trans*-spliced. A comparative analysis with other plant mt genomes showed that four syntenic gene clusters were conserved in all plant mtDNAs. Fifteen chloroplast-derived fragments were strongly associated with mt genes, including the intact sequences of the chloroplast genes *psaA, ndhB* and *rps7*. According to the plant mt genome evolution analysis, some ribosomal protein genes and succinate dehydrogenase genes were frequently lost during the evolution of angiosperms. Our data suggest that the kenaf mt genome retained evolutionarily conserved characteristics. Overall, the complete sequencing of the kenaf mt genome provides additional information and enhances our better understanding of mt genomic evolution across angiosperms.

## Introduction

Mitochondria are the main organelles responsible for plant energy metabolism and play an imperative role in supplying ATP via oxidative phosphorylation during development, reproduction and various biochemical processes in plants. According to endosymbiotic theory, plant mitochondria are thought to be descended from free-living bacteria, which explains the presence of their genomes^1^. The structure of the plant mitochondrial (mt) genome has undergone dramatic changes over long-term evolution. Horizontal transfer with frequent exchanges among the nucleus, plastids and mitochondria appears to be responsible for the acquisition of exogenous sequences^2^. In addition, the abundance of repeated sequences of various sizes and numbers is involved in mt genome homogeneous recombination^3^. Thus, the noncoding regions vary and exhibit low conservation across species, which renders the sequencing of plant mt genomes, particularly in angiosperms, extraordinarily difficult. The first report of an angiosperm mt genome was achieved in *Arabidopsis thaliana*^4^. With recent sequencing efforts over the past decade, the mitochondria of many angiosperm species (e.g., *Beta vulgaris*^5^, *Oryza sativa*^6^, *Brassica napus*^7^, *Zea mays*^8^*, Triticum aestivum*^9^*, Nicotiana tabacum*^10^, *Vitis vinifera*^11^*, Citrullus lanatus*^12^, *Vigna radiata*^3^, *Cucumis melo*^13^, *Gossypium hirsutum*^14,15^ and other higher plants^16–19^) have been sequenced. DNA sequencing and physical mapping have been used to identify several evolutionarily conserved properties of plant mt genomes, i.e., gene order, genome structure, and migration of sequences from other organelles.

Angiosperm mt genomes are complex and vary substantially in size, ranging from 208 kb in *Brassica hirta*^5^ to 11.3 Mb in *Silene conica*^20^. Despite the great variation in size and physical mapping properties, plant mitochondria exhibit significant conservation in functional genes, including 37–83 protein coding, tRNA and rRNA genes^21^. The shuffling of mtDNA sequences by recombination, repeat sequences and most noncoding sequences plays an important role in mt genome evolution by changing the gene organization and creating chimeric genes^22,23^. In most plant mt genomes, many homologous sequences are derived from the chloroplasts and nucleus^6,9^. In *Cucumis melo* mt genomes, 35 DNA fragments were found to originate from the chloroplast genome, while 1,114 DNA fragments with a total length of 1,272.6 kb were homologous with the nuclear genome, accounting for 46.5% of the mt genome^13^. Furthermore, horizontal gene (or DNA) transfers appear to be responsible for the integration of exogenous DNA and explain the complex structure of angiosperm mt genomes^24,25^.

Kenaf (*Hibiscus cannabinus*) is an important fibre crop that is widely used in paper-making and weaving^26^. However, data regarding the mt genome sequence of kenaf are limited. Here we report the first complete kenaf mt genome of UG93B, which was a maintainer line and derived from the wild type of UG93. In the present study, the structure of first the complete kenaf mt genome sequence was determined, and phylogenetic analyses were performed for comparisons with angiosperm mt genomes. Our data provide basic information and a better understanding of the evolutionary processes of kenaf mt genome.

## Results

### Kenaf mitochondrial genome sequencing and assembly

**Figure 1:**
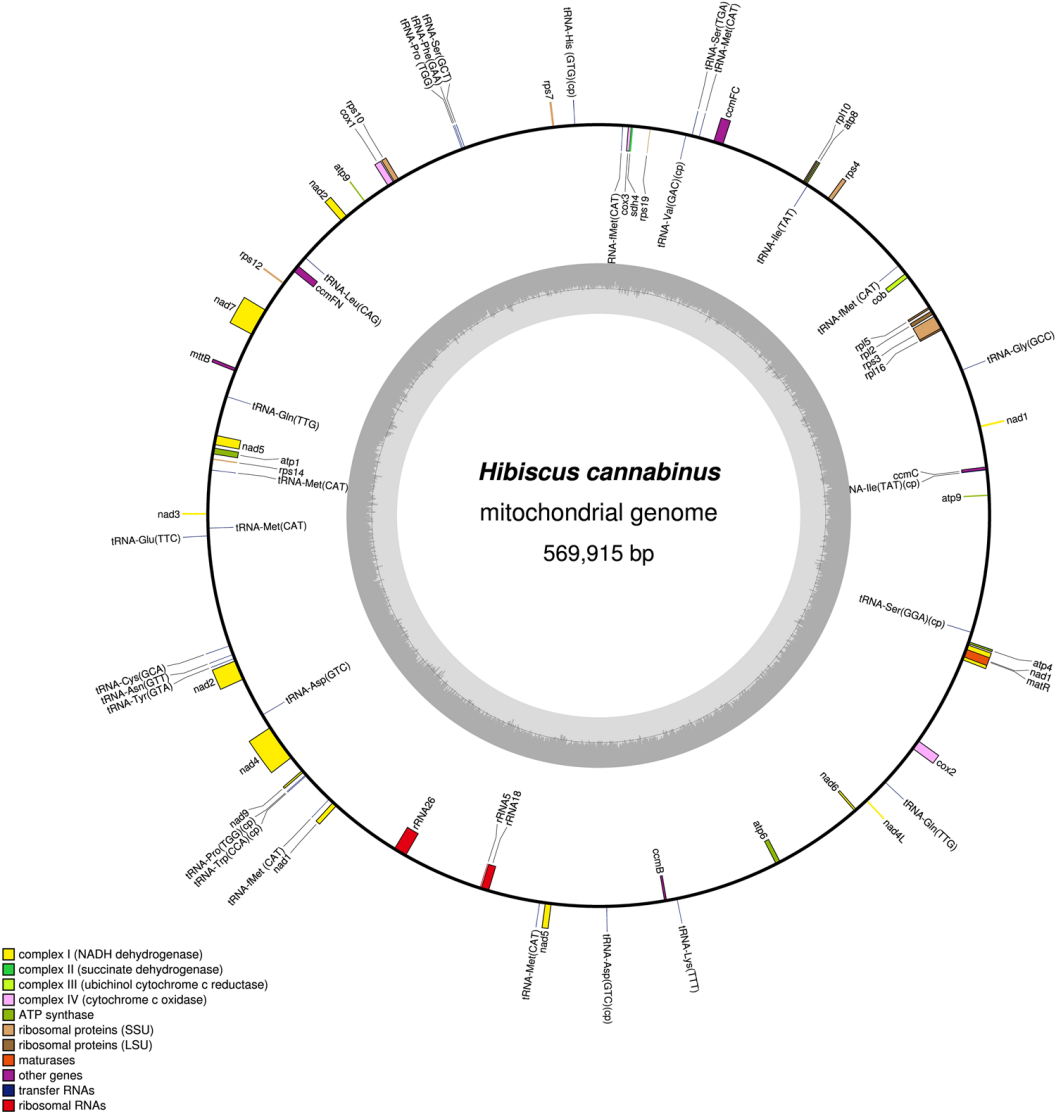
Map of the *Hibiscus cannabinus* (kenaf) mt genome.

Isolated kenaf mitochondrial DNA (mtDNA) was used to construct a library for sequencing using PacBio RS II single-molecule real-time sequencing technology (SMRT), which generated 1.12 G of raw data, with an average read length of 4.6 kb, and the longest read was 32 kb. In total 67,152 reads (363,717,023 bp) were obtained after removing the adapter and low-quality regions, and the average coverage depth, read length and read quality were 605×, 5.4 kb, and 0.81, respectively (Supplementary Table S1). In total 1,819 reads (12,114, 267 bp, average length of 6.7 kb) were obtained after correcting by mapping the short reads to the long seed reads. After filtering the chloroplast reads, 1,762 reads (11,733,852 bp, average length of 6.7 kb) were used for the assembly process. Finally, the kenaf mt genome was assembled into a single circular molecule with a total length of 569,915 bp and an overall GC content of 44.9% (Fig. 1, Supplementary Table S2).

### Gene content in kenaf

**Figure 2:**
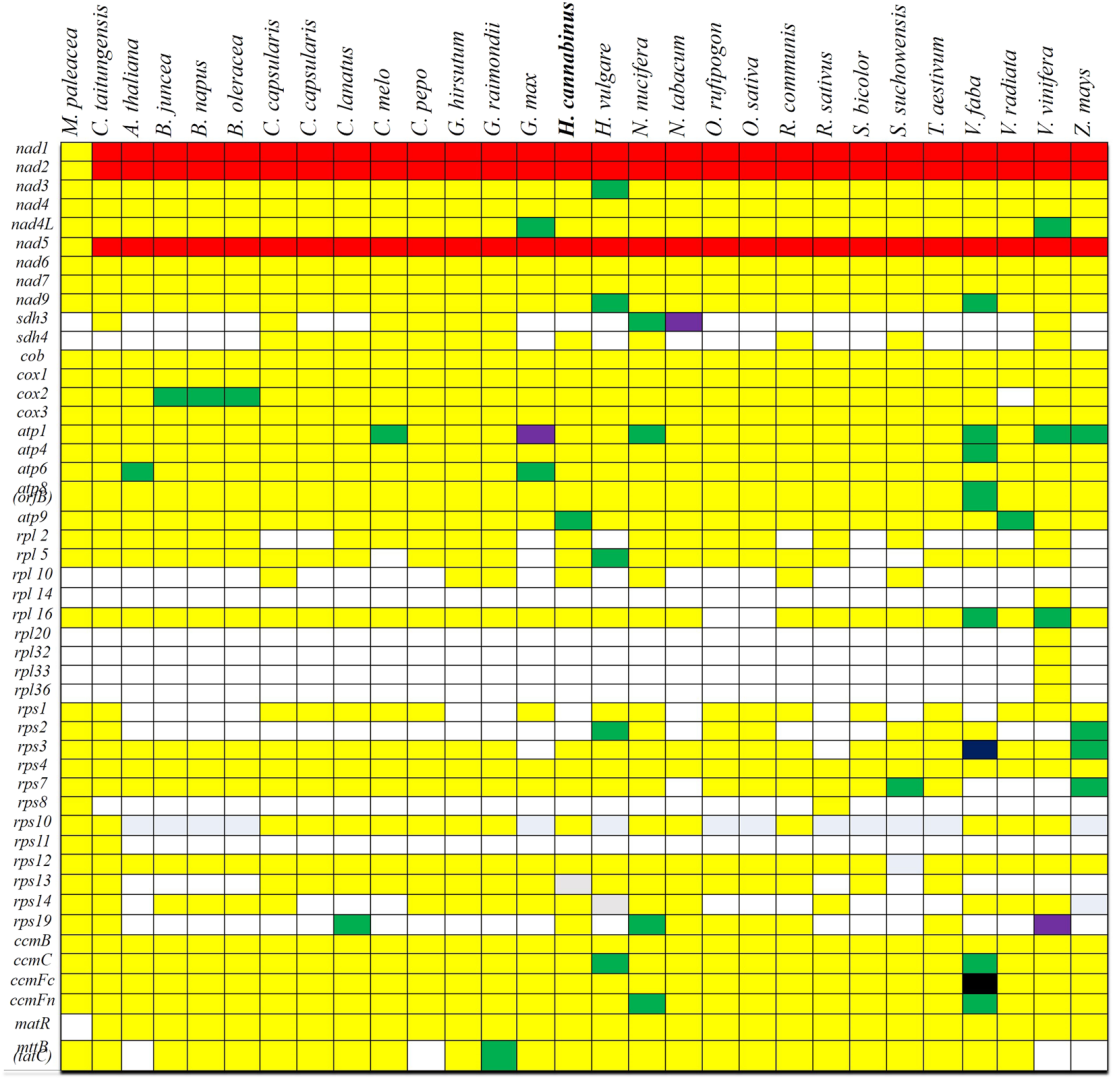
Distribution of protein-coding genes in plant mitochondrial genomes. White boxes indicate that the gene is not present in the mt genome. Yellow, green, purple, blue and black boxes indicate that one, two, three, four and six copies exist in the particular mt genome, respectively. Red boxes indicate trans-splicing. Kenaf (*Hibiscus cannabinus*) is shown in bold.

Sixty-two genes including 36 core protein-coding genes, conserved among all plant mt genomes were annotated by comparing the assembled kenaf mt sequence with known plant mt sequences in the NCBI public DNA database using BLASTn. The kenaf mt genome contains 20, 7, 4, 4, 3, 29, 1 and 1 genes responsible for electron transport, oxidative phosphorylation, small ribosomal proteins, large ribosomal proteins, cytochrome C maturation protein, rRNAs, tRNAs, and *matR* and *mttB*, respectively (Supplementary Table S3). Most protein-coding genes, except for *sdh3*, *rps13* and *rps19*, were identical to those in the mt genome of the *Gossypium* species (Fig. 2). Twenty-three tRNA genes specifying 18 amino acids were identified in the kenaf mt genome. Of these genes, 15 tRNAs had a mt origin, and eight tRNA had a chloroplast origin (Table S3). The presence and locations of these genes in the kenaf mt genome and comparisons with other plant mt genomes are shown in Fig. 1 and Supplementary Table S4.

In most spermatophytes, the genes responsible for the electron transport chain and oxidative phosphorylation are conserved, except for mitochondrial complex II, which contains *sdh3* and *sdh4*. Notably, the high diversity in the gene content among the higher plant mt genomes was a primary contributor to the variety of ribosomal protein genes (Fig. 2). The mt genome of plants is known to contain genes encoding products involved in electron transport, oxidative phosphorylation, ATP synthesis, cytochrome c biogenesis, ribosomes, and the translation of proteins(Fig. 2).

The protein-coding genes in the kenaf mt genome account for 6.9% of the genome and a total length of 39,534 bp. In addition, 126 open reading frames (ORFs) larger than 100 amino-acid residues in size were annotated in the kenaf mt genome (Supplementary Table S2). However, none of these ORFs could be assigned a function based on sequence similarity at either the nucleotide or protein level. Most of ORFs were considered hypothetical proteins. A putative protein of 295 amino-acid residues encoded by ORF295 has unknown functions, although its first 183 nucleotides were similar to those of *rps4* from the 5′ to 3′ end (Supplementary Fig. S2). The nucleotide sequence in the coding region had no similarity to any other plant mt genomes, except for a part of the *Gossypium* mt genome. This chimeric characteristic of ORF295 may have resulted from a horizontal gene transfer (HGT) event between angiosperm mt genomes. Interestingly, despite the large size differences among the mt genomes of various higher plant species, these genomes share a similar set of functional genes (protein, rRNA and tRNA genes), which is consistent with the results reported by Mower *et al*.^27^. However, the additional ORF e.g., ORFs identified in the kenaf mt genome, were not shared even among closely related plants, suggesting that many ORFs likely do not encode functional proteins and may have unidentified species-specific functions.

### Repeat sequences of kenaf mitochondrial DNA

Repeat sequences are extensively found in the plant mt genome, are characterized primarily as forward repeat and palindromic repeats, and exhibit high levels of polymorphism. In the present study, we identified 584 repeat sequences that ranged from 20 to 7,782 bp and accounted for 11.71% of the total kenaf mt genome (Supplementary Table S2, Fig. S3). Most repeats (approximately 95%) were between 20 and 100 bp in length, accounting for 6.63% of the total genome; approximately 5% (28) of the repeats were larger than 100 bp, and three repeats were larger than 1 kb (R1, 7,782 bp; R2, 1, 877 bp; and R3 1,528 bp) (Table 1). Most repeat sequences (≥60 bp) contained 2 copies of the repeat, and eight repeat sequences contained three copies (Table 1).

**Table 1:** Repeats (≥60 bp) in the kenaf mt genome.

No.	Size (bp)	Identity (%)	Copy-1		Copy-2^a^		Copy-3^a^		Type^b^
			Start	End	Start	End	Start	End	
R1	7782	100	814	8595	196628	188847			P
R2	1877	100	62808	64684	356837	354961			P
R3	1525	100	60919	62443	446303	444779			P
R4	842	100	204427	205268	367743	384565			F
R5	535	100	62438	63272	445775	444941			P
R6	468	100	287766	288233	413387	412920			P
R7	433	100	62845	63277	136700	137132	358244	357812	F/P
R8	394	100	431948	432341	569460	569853			F
R9	374	100	4933	5306	199917	199544	380908	381281	P/F
R10	229	100	237021	237249	320344	320572			F
R11	210	100	28517	28726	243614	243405			P
R12	204	100	84633	84836	413895	414098			F
R13	190	100	326	515	204717	204528	368033	367844	P
R14	181	100	284825	284645	539224	539404			F
R15	174	100	73673	73846	539296	539469			F
R16	165	100	358549	358713	445775	445939			F
R17	146	100	247536	247681	510485	510630			F
R18	137	100	102129	102265	458421	458557			F
R19	135	100	683	817	204407	204273			P
R20	128	100	136700	136827	445775	445648			P
R21	115	100	683	797	367743	367629			P
R22	109	100	45409	45517	456623	456731			F
R23	109	100	73673	73781	284897	285005			F
R24	107	100	85072	85178	273126	273232			F
R25	103	100	176182	17714	380789	380891			F
R26	97	100	47563	47659	538666	538762			F
R27	91	100	362171	362261	532778	532868			P
R28	88	100	34694	34781	258881	258968			F
R29	88	100	102046	102133	137837	137924			P
R30	88	100	140828	140915	465024	465111			P
R31	86	100	62665	62750	268392	268477	445997	446082	F/ P
R32	82	100	31305	31386	301188	301269			F
R33	81	100	368575	368655	456163	456243			F
R34	80	100	4243	4322	200901	200980	268607	268686	P /F
R35	77	100	28446	28522	243819	243895			P
R36	75	100	333330	333404	429353	429427			P
R37	74	100	11547	11620	134973	135046			F
R38	73	100	41569	41641	438195	438267			F
R39	73	100	268577	268647	539445	539517			F
R40	70	100	189	258	204978	205047	368294	368373	P
R41	66	100	141384	141449	452383	452448			P
R42	65	100	4873	4937	200286	200350	380849	380913	P/F
R43	65	100	200286		380852				P
R44	63	100	141511	141573	452513	452575			F
R45	63	100	41704	41766	438338	438400			F
R46	61	100	47590	475650	275165	275225	538696	538756	P
R47	61	100	432343	432403	569854	569914			F
R48	61	100	73792	73852	285008	285068			F

^a^Compare with copy-1 as control. ^b^The letters F and P represent forward repeats and palindromic repeats, respectively. The numbers listed in the starting and ending points refer to positions in the kenaf mt genome sequence (GenBank accession MF163174).

### Introns

In the kenaf mt genome, nine mt genes, composed of 25 introns ranging from 41 to 2,878 bp in size were identified, and occupied 7.8% of the total kenaf mt genome (Supplementary Table S4). Five of the nine mt genes were *nad1, nad2, nad4, nad5* and *nad7*, which are components of mitochondrial complex I, and the remaining four genes were *cox2*, *ccmFc*, *rps3* and *rps10*. In addition, five *trans*-spliced introns observed in *nad1, nad2* and *nad5* were fragmented into separate coding regions, which is consistent with angiosperm plants. Twenty *cis*-spliced intron sequences were observed in the remaining mt genes. The intron locations and splicing were highly similar to those observed in other higher plant mt genomes (Fig. 2).

### Chloroplast-like sequences

BLASTn was used to identify chloroplast-like sequences in the kenaf mt genome. Twelve such sequence fragments were identified and showed >97% nucleotide sequence identity with the corresponding chloroplast sequences, and the segments ranged from 73 bp to 2,653 bp with a total length of 11,281 bp (accounting for 1.98% of the genome size). These chloroplast-derived fragments included eight tRNA-related sequences. Moreover, three intact chloroplast-related genes, i.e., *ndhB*, *psaA* and *rps7*, were identified in the kenaf mt genome (Supplementary Table S5).

### Gene organization and gene clusters in plant mt genomes

**Figure 3:**
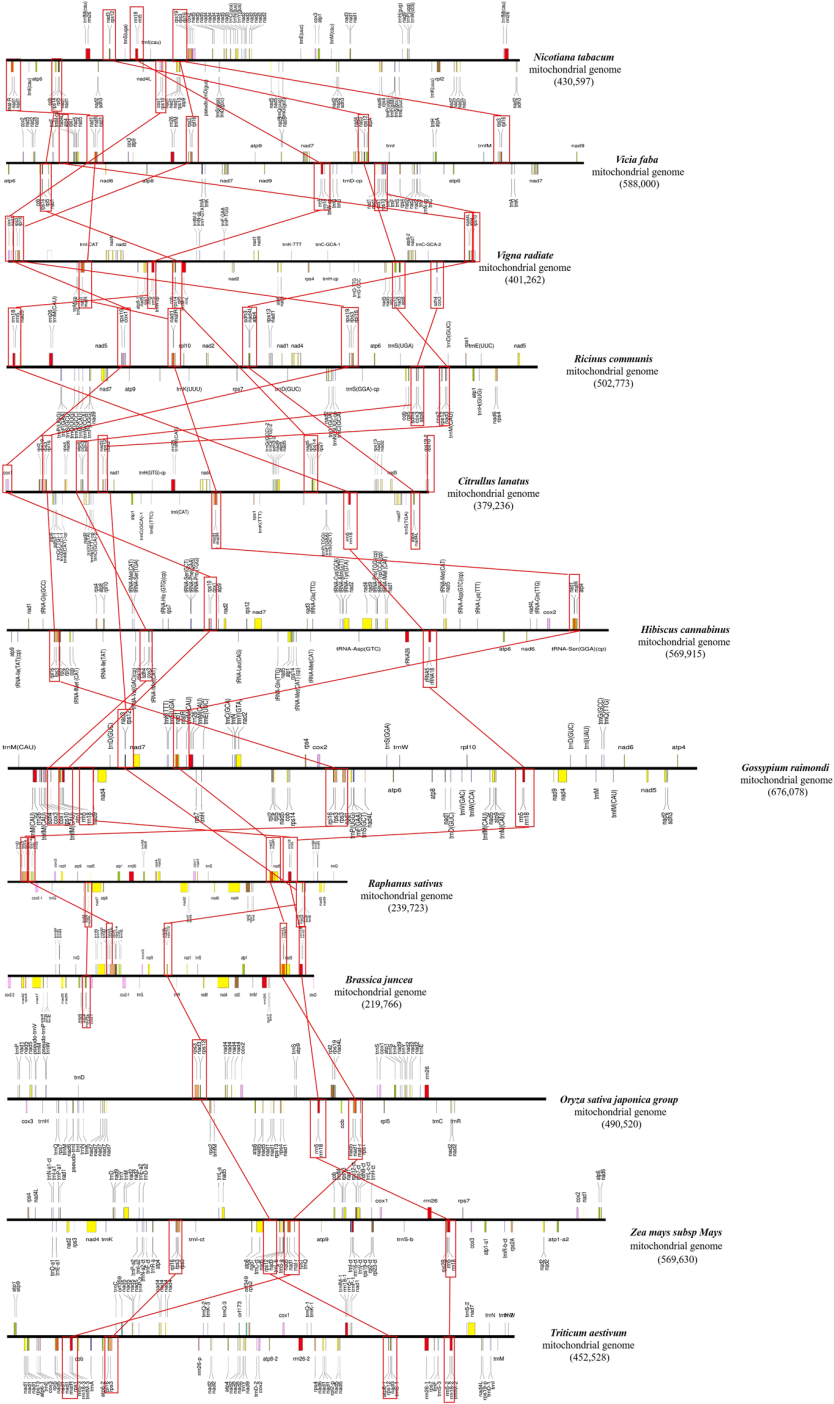
Analysis of conservative gene clusters between the kenaf mt genome and other higher plant mt genomes.

The gene organization greatly differs among plant mt genomes. In this study, we compared the gene orders in the 28 mt genomes and counted the number of syntenic gene clusters (genes that remain in the same order). Four gene clusters (i.e., *rrn5-rrn18, nad1-matR*, *rps12-nad3*, and *rps3-rpl16*) were found to be highly conserved in the plant mt genomes (Fig. 3, Supplementary Table S6). The gene cluster *cox3-sdh4* was widely distributed in most dicotyledonous species, except for *Brassicaceae*, while the conserved *rpl5-rps14* gene cluster was scattered in the other dicotyledonous species, but present in all *Brassica* spp. Understandably, species that have close evolutionary relationships share more clusters. Each gene cluster is transcribed from the same strand, implying that the genes may undergo co-transcription as a polycistronic mRNA.

### Distribution of tRNAs and DNA transfer from the plastid to mitochondrial DNA

**Figure 4:**
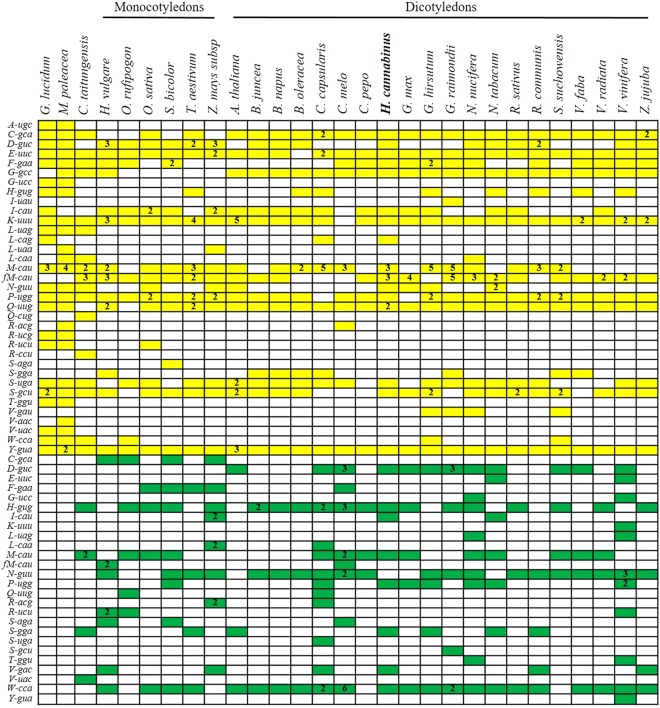
tRNA distribution map in plant mitochondrial genomes. Yellow boxes and green boxes represent mt tRNA genes and chloroplast-like tRNA genes with one copy in plant mtDNA, respectively. The numbers in the cells represent the copy numbers in the plant mtDNA. Blank boxes indicate that the tRNA gene is absent.

A complete set of tRNAs is essential for protein translation in the plant mt genome. However, many tRNAs undergo loss, migration and inactivation during mt genome evolution in higher plants^27^. To evaluate the origin and distribution of the tRNA genes, tRNA scan-SE (http://lowelab.ucsc.edu/tRNAscan-SE/) was used to predict the number and types of tRNA genes in the kenaf mt genome. In total, 23 tRNA genes were identified, and these genes recognized 18 amino acids (i.e., Asp, Gly, Met, Ser, His, Phe, Pro, Glu, Cys, Asn, Tyr, Trp, Asp, Lys, Ser, Leu, Ile, and Val). Thus, tRNA genes for two amino acids (i.e., Ala and Thr) were not identified and appeared to be missing from the kenaf mt genome (Fig. 4). Of these 23 tRNAs, eight had a plastid origin, and twenty-one had a mt origin.

The mt genomes of twenty-eight land plants and the fungal species *G. lucidum* were analysed to explore the patterns of tRNA loss during the evolution of plant mt genomes. Only *G. lucidum* has a complete set of tRNAs (Fig. 4). The *trnA* gene was lost from gymnosperms to angiosperms, indicating that *trnA* was lost early in the evolution of land plants. The *trnG* gene was absent from monocots, but existed in dicotyledons, suggesting that this gene was specifically present in dicotyledons. Although *trnL, trnR, trnT* and *trnV* were lost during the evolution of angiosperms, *trnR* and *trnV* existed in certain dicotyledons, suggesting that these genes may have been subsequently regained. Interestingly, most of the tRNAs in *C. melo* exhibited a pattern of plastid-like origin, suggesting that frequent exchanges occurred between the mt genome and the chloroplast genome.

BLASTn was used to assess the mt sequence fragments that originated in the chloroplast. Four chloroplast-derived fragments (*trnH, trnM, trnN* and *trnW*) were found to be conserved in all analysed mt genomes, and one (*trnD*) and two (*trnC* and *trnF*) chloroplast-derived fragments were found to be conserved in dicots and monocots, respectively. In contrast, other chloroplast-like tRNA genes exhibited scattered distributions, and certain native tRNA genes were irregularly lost among the higher plant mt genomes, suggesting that the gain and loss events of the tRNA genes occurred multiple times during evolution. Overall, *trnC, trnE, trnK*, *trnM, trnP, trnQ, trnS* and *trnY* were present in all species evaluated, indicating that these tRNAs are highly conserved in plant mt genomes.

### Conserved sequences and phylogenetic analysis

**Figure 5:**
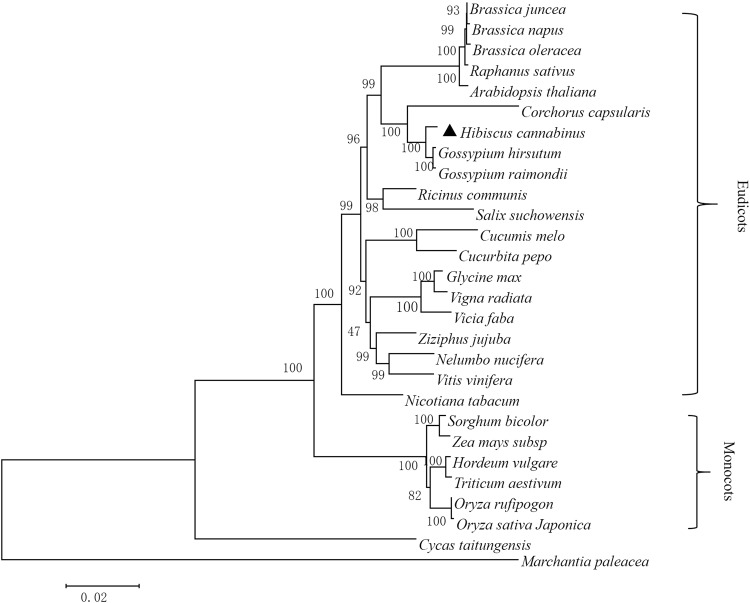
The original phylogenetic tree of 22 functionally related genes. The genes used are listed in Table S2 and include 17 respiratory complex genes, four cytochrome c biogenesis genes and a *cob* gene, and the tree was rooted with *Marchantia paleacea*.

**Figure 6:**
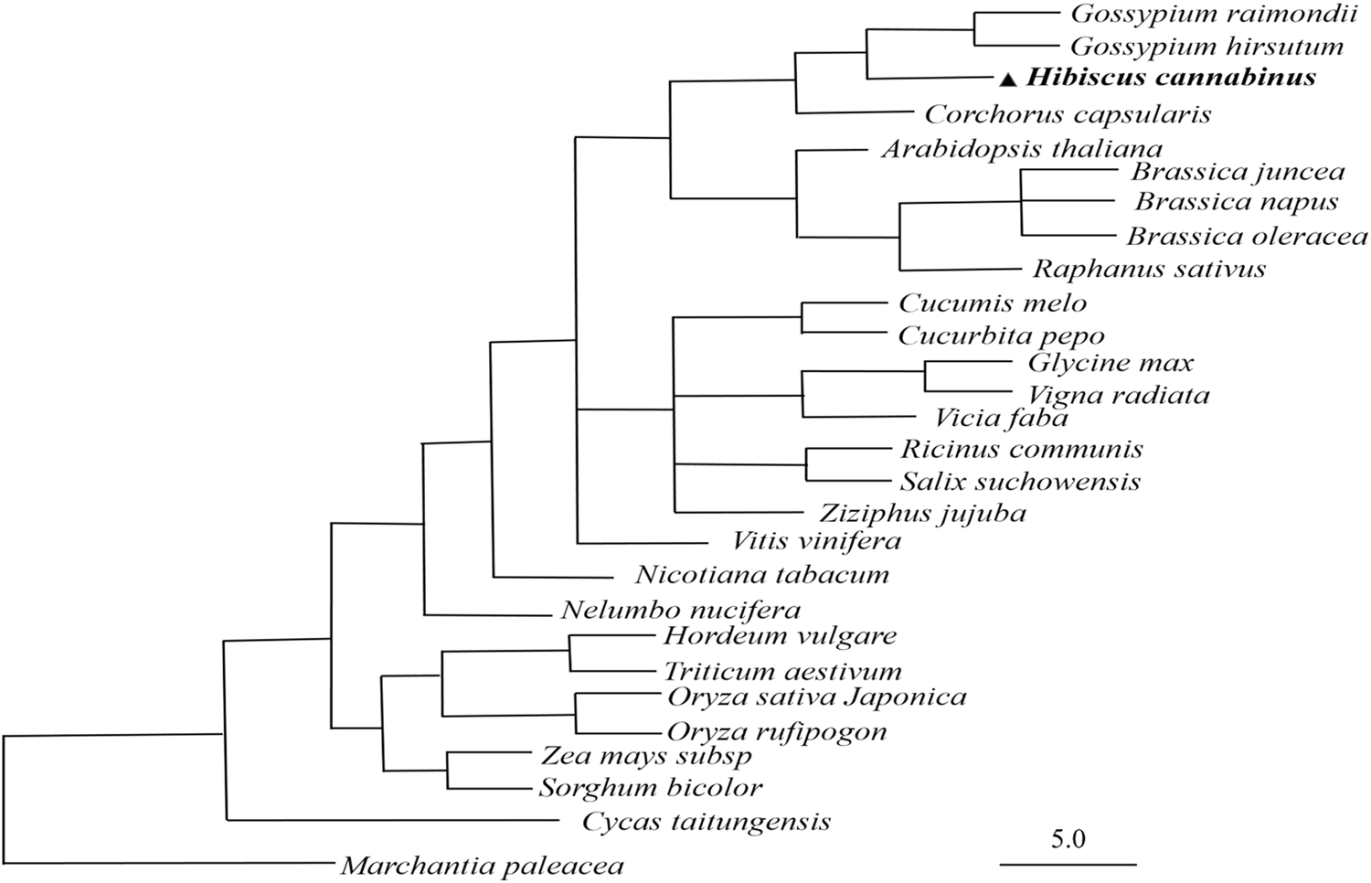
NCBI taxonomy common tree of 28 analysed species.

A phylogenetic analysis was performed to determine the evolutionary relationships among the mt genomes of twenty-eight plant species, included angiosperms and gymnosperms, and bryophytes was chosen as the outgroup. The chloroplast-derived sequences and non-protein-coding sequences were removed before blasting against the other mt genomes. First, these mt functional genes were concatenated in a head-to-tail format. Maximum likelihood method was used to complete the phylogenetic tree analysis. As shown in Fig. 5, the *Hibiscus cannabinus* and *Gossypium* species belonging to the *Malvaceae* family were classified into one clade with a high bootstrap support value of 100. In addition, the species share a high sequence similarity, as supported by the higher bootstrap support values. Species belonging to different groups share less sequence similarity and have reduced bootstrap support value. The phylogenetic tree strongly supported the separation of monocot plants and dicot plants, and the separation of angiosperms from gymnosperms. Additionally, the evolutionary relationship of these 28 plant species was analysed using the plant taxonomy method and used to construct an NCBI taxonomy common tree (Fig. 6). The phylogenetic relationships based on mt genome homologous sequences are consistent with the species taxonomy despite the exceptional variability among these mt genomes.

To further explore the utility of these mt genes in phylogenetic reconstruction, twenty-two mt genes were divided into five groups according to the function of their proteins (Supplementary Table S3), and the genes in each group were assembled in a head-to-tail arrangement. Among the five groups of phylogenetic trees, the set of mitochondrial complex I genes was congruent with a previous reconstruction based on 22 functionally related genes (Supplementary Fig. S4). The trees of mitochondrial complex III and complex IV reconstructing the divergence of monocots and dicots resulted in topologies that differed from those obtained by the previous reconstruction base on 22 functionally related genes, but the species fell into clades that belonged to the same family (Supplementary Figs S5, S6). The phylogenetic trees of the mitochondrial complex V and cytochrome c biogenesis genes revealed evolution relationships that slightly differed from those obtained with the previous reconstruction base on 22 functional related genes (Supplementary Figs S7, S8). Therefore, the phylogenetic analysis base on the function of mitochondrial genes revealed slightly different topologies, but the species fell into clades that were consistent with their family designations. In contrast, the phylogenetic tree based on the whole set of genes was congruent with the species taxonomic tree (Fig. 6).

## Discussion

Third-generation SMRT sequencing technology based on the PacBio RS II platform can produce substantially longer reads (>5 k/read) than second generation sequencing^28^. Which also can be used to closed genome gaps, whole-genome sequencing projects for any species^29–35^ and non-SNP DNA variations identification^36^. In our study, the SMRT sequencing technology was used to sequence the whole mt genome sequence of kenaf (*Hibiscus cannabinus)*. We obtained the kenaf mt sequence with a high accuracy, and the genome size was 569,915 bp. The longest read was 32 kb, which is much longer than the usual reads obtained using other sequencing technologies.

### Characteristics of plant mitochondrial genes

Plant mtDNAs primarily comprise of protein-coding genes, tRNAs and rRNAs. The kenaf mt genes included only 36 of the 41 protein-coding genes present in ancestral land plant mt genomes^27^, indicating that several protein-coding genes were lost or transferred to other organelles during the evolution of kenaf mitochondria. The frequent loss and functional transfer of ribosomal protein genes and succinate dehydrogenase (*sdh*) genes to the nuclear genome were the main causes of the variable gene contents among the plant mt genomes. This finding is consistent with previous results that have been confirmed by Southern blot hybridization^21^. Our results revealed the presence of only *sdh4* in the kenaf mt genome, while both *sdh3* and *sdh4* were identified in the closely related *Gossypium* species^37^. Thus, the presence of the succinate dehydrogenase genes is highly variable, even among evolutionary close angiosperm species. However, two *atp9* copies were identified in the kenaf mt genome, which may have resulted from HGT events or mtDNA recombination during the evolution of the kenaf mt genome.

### Repeat sequences in the genome

The mt genomes of land plants, particularly angiosperms, are frequently characterized by repeat sequences^38^, which could explain most of the variation in the mt genome size. Moreover, these sequences are sites of intragenomic recombination, underlining the evolutionary changes in the mt genome organization in *vivo*^39,40^. Tandem simple and scattered repeat sequences are extensively found across plant mt genomes and exhibit high levels of polymorphisms^41,42^. The repetitive sequences in the *Cucumis melo* mt genome had a size of 2,738 kb and comprised primarily small repeats, accounting for 42.7% of the mt DNA^13^. In contrast, other genomes contain fewer larger segmental duplications^41,43^. The *Vitis* mt genome (773 kb) has only 6.8% repetitive DNA sequences^11^, while the moderately sized Legume *vign*a genome (401 kb) has fewer and smaller repeats that account for 2.7% of the mt genome. These data suggest that the genome size is not a good indicator of repeat content in angiosperm mt genomes. In the present study, the repetitive structure of the kenaf mt genome accounted for 11.71% of the genome. These sequences are poorly conserved across species and have a high proportion of smaller repeats, indicating that the increased size of the kenaf mt genome was primarily due to the duplication of short sequences.

### Introns in the mitochondrial genome

Land plant mt genomes contain a large and variable number of introns, ranging from 19 in *Silene latifolia*^44^ to 34 in the hornwort *Phaeoceros laevis*^45^. According to the present study, the kenaf mt genome retained 25 introns, disrupting 9 protein genes, which is consistent with the common ancestor theory of gymnosperms and angiosperms^27^ and suggests that the introns were lost or gained during plant evolution. However, *cis*-splicing is ubiquitous in most introns of seed plants, while the mt genes *nad1, nad2* and *nad5* evolved a split structure that requires *trans*-splicing and were highly consistent with the sequenced angiosperm mt genomes^27^. Thus, the transcription process of introns was conserved among the angiosperm mt genomes.

### Conservation of gene clusters

Genome recombination can disrupt clusters, while multiple recombination events can generate similar syntenic gene clusters, leading to vast differences in the gene order among plant mt genomes^46^. In general, evolutionarily close species have more similar gene orders and clusters. Two gene clusters (*rrn18-rrn5* and *nad5-nad1-matR*) are conserved in all land plant mt genomes, and may date back to the original plant mt genomes of liverworts, mosses, and most charophytes^27^. In contrast, the *rps12-nad3* and *(rps19)-rps3-rpl16* gene clusters were evolutionary conserved in most land plant mt genomes but absent from *M. paleacea* and *S. suchowensis*, respectively^47,48^. Two other gene clusters (*rps10-cox1* and *sdh4-cox3-(atp8)*) were specifically conserved in dicots, except for *Brassica*^49^. The cluster of *atp4-nad4L* exists in all surveyed dicots, except for the species of *Gossypium*, *H.cannabinus* and *C. capsularis*. These exceptions were likely due to frequent recombination events during plant mt genome evolution. In our study, the characteristics of the gene clusters in the kenaf mt genome were consistent with the general conservation of most dicotyledon, indicating that the gene clusters in the kenaf mt genome were more conserved during plant mt genome evolution.

### DNA transfer in the mitochondrial genome

HGT is thought to be the main process during the acquisition of exogenous sequences^50–52^. The transfer of DNA sequences among plastid, nuclear and mt genomes is a common phenomenon that has been observed in the fully sequenced mt genomes of land plants^53,54^. Although the amount of plastid DNA in the mt genome is 3–6% in most examined species, plastid DNA varies from 2 kb (0.5%) in the Legume *Vigna* to 113 kb (11.5%) in *Cucurbita pepo*^12^. In many cases, these plastid-to-mitochondrion transfers have resulted in the insertion of plastid genes into the mt genome, but most genes are clearly nonfunctional^53^. Occasionally, the only plastid genes that are transferred into the mt genome and remain functional encode tRNAs. The absence of chloroplast-derived tRNAs from liverworts, moss, hornworts, and bryophyte*s* indicates that DNA transfer from the chloroplast genome to the mt genome might have occurred after the divergence of gymnosperms and angiosperms. Eight chloroplast-derived tRNAs identified in the kenaf mt genome can be traced to the retention of an earlier HGT event.

The plastid-derived *trnW* (GTT) and *trnH* (GTG) genes are frequently observed in angiosperms but are absent from *C. taitungensis*, indicating that these tRNA genes may have been transferred after the separation of angiosperms. Identifying the numbers and types of tRNA genes in the kenaf mt genome may be helpful for evaluating the origin and evolution of tRNA genes in higher plants. These results suggest that the intracellular transfer of tRNA and ribosomal genes from the chloroplast to the mitochondria was a frequent process.

## Conclusion

Plant mt genomes are intriguing due to their highly conserved genic content and slow rate of genic evolution. In contrast, features, such as the genomic structure, the genome size and repeat sequences, are highly variable. In this study, we determined the complete sequence of the kenaf mt genome. The comparison of the kenaf mt genomic features with those of other plant mt genomes should provided a more comprehensive understanding of mt genome evolution in higher plants. The complete mt genome of kenaf shares many common genomic characteristics with other plant mt genomes, such as the conservation of genic content, gene clusters, certain intergenic sequences and tRNA gene origin and distribution. These observations suggest that the evolution of mt genomes is consistent with the species relationships in plant taxonomy. However, the highly dynamic genome structures (genome size and gene order) suggest that the recombination of higher plant mt genomes is independent and random among species. The sequencing of the kenaf mt genome contributes to our understanding of the characteristics of the mt genome across angiosperm evolution.

## Materials and Methods

### Mitochondrial DNA isolation and sequencing

Mitochondria were isolated from the kenaf maintainer line UG93B and purified from 7-day-old etiolated seedlings using differential centrifugation and discontinuous (18%, 23% and 40%) Percoll density gradient centrifugation according to the methods described by Wilson and Chourey^55^. The mitochondrial DNA isolation was performed as described by Sue^56^ with modifications. The purified mitochondria were lysed with cetyltrimethylammonium bromide (CTAB) supplemented with 2% polyvinylpyrrolidone and 0.7% β-mercaptoethanol (Solarbio, Beijing) at 65 °C for 30 min. The lysis solution was extracted two to three times with chloroform/isoamyl alcohol (24:1), and absolute ethyl alcohol was used to precipitate the mtDNA. DNase-free water (50 μL) was added to resuspend the DNA pellets. The integrity, quality and concentration of the UG93B mtDNA were analysed using agarose gel electrophoresis, a NanoDrop 2000 (Thermo Scientific, USA) and a Qubit fluorometer (Thermo Scientific, USA).

### DNA sequencing and genome assembly

In total, 20 μg of UG93B mtDNA were randomly sheared to fragments using a Covaris S220 (Thermo Scientific, USA). Large fragments with an average size of 20 kb were purified by magnetic bead enrichment. SMRTbell templates were obtained by ligating the hairpin adaptors to the end of a double-stranded DNA molecule and removing the failed ligation products with exonuclease. An Agilent 2100 Bioanalyzer High Sensitivity Kit (Agilent Technologies, USA) was used to assess the quality of the library. Subsequently, eight SMRT cells were sequenced using P4-C2 reagents on a PacBio RS II sequencing platform^57^. The sequencing and *de novo* assembly were performed at Nextomics Biosciences Co., Ltd, Wuhan, China. The clean reads were obtained by filtering out the sequencing adapters and low-quality sequences using SMRT Analysis 2.3.0 with the default settings. The kenaf mt genome sequence was extracted from filtered reads containing both chloroplast and mt genomes. Blat^58^ was used with the default parameters against the NCBI chloroplast genome data to filter reads containing chloroplast genomes, and reads with a match greater more than 90% were moved. The kenaf mt genome sequence was assembled using a Hierarchical Genome Assembly Process (HGAP) workflow, including preassembly, error correction, Celera assembly and polishing using Quiver^59^. The long reads were selected as “seed” reads, to recruit all other subreads and construct highly accurate preassembled reads using a directed acyclic graph-based consensus procedure. This procedure was followed by assembly using off-the-shelf long-read assemblers. A basic local alignment with successive refinement (BLASR) was used to align the short reads to the seed reads and improve the sequence accuracy^60^. The Celera assembler software and overlap-layout-consensus (OLC) algorithm were used to assemble all corrected contigs^61^. Finally, Quiver was used to improve the site-specific consensus and generate the gap-free kenaf mt genome. Additionally, a specific prime pair was designed to verify the circular mitochondrial genome of kenaf (Supplementary Fig. S1).

### Genome annotations and analyses

The mt genomes were annotated using BLASTn and MITOFY^12^ using previous angiosperm mt genes to query sequences in the NCBI database (https://www.blast.ncbi.nlm.nih.gov). The tRNA genes were identified using the tRNA scan-SE software (http://lowelab.ucsc.edu/tRNAscan-SE/). ORFs that contained more than 100 amino-acid residues and started with methionine were predicted and annotated using ORF-Finder (http://www.ncbi.nlm.nih.gov/gorf/gorf.html). The repeat sequences were analysed using REPuter software (http://bibiserv.techfak.uni-bielefeld.de/reputer) with the following parameters: the repeat sequence was at least 20 bp in length and the repeat identity was greater than 90%^62^. The circular map and syntenic gene cluster maps of the plant mt genomes were created using OGDRAW v1.2 (http://ogdraw.mpimp-golm.mpg.de/)^63^. The annotated genome sequence was submitted to NCBI under the GenBank accession no. MF163174.

### Phylogenetic analysis

To compare the kenaf mt genome to other plant mt genomes, 28 plant mt genomes, including *Arabidopsis thaliana* (NC_001284), *Brassica juncea* (JF920288), *Brassica napus* (KP161618), *Brassica oleracea* (JF920286), *Brassica oleracea* (AP012988), *Cycas taitungensis* (NC_010303), *Citrullus lanatus* (NC_014043), *Glycine max* (JX463295), *Cucumis melo* (JF412792), *Cucurbita pepo* (NC_014050), *Gossypium raimondii* (KU317325), *Gossypium hirsutum* (JX065074), *Hordeum vulgare subsp* (AP017301)*, Marchantia paleacea* (NC_001660), *Nelumbo nucifera* (KR610474)*, Nicotiana tabacum* (BA000042), *Oryza rufipogon* (AP011076), *Oryza sativa japonica* (BA000029), *Raphanus sativus (JQ083668), Ricinus communis* (HQ874649), *Salix suchowensis* (NC_029317*), Sorghum bicolor* (DQ 984518), *Triticum aestivum* (AP008982), *Vicia faba* (KC189947), *Vigna radiate* (NC_015121), *Vitis vinifera* (NC_012119), *Zea mays subsp. mays* (NC_007982), and *Ziziphus jujuba* (KU187967), were downloaded from the NCBI Organelle Genome Resources database (http://www.ncbi.nlm.nih.gov/genome/organelle/). These mt genome sequences were selected because they are available for analysis in NCBI and are clearly taxonomically classified. Phylogenetic analyses were performed using concatenated exon sequences from 22 conserved protein-coding genes (*atp1, atp4, atp6, atp8, atp9, ccmB, ccmC, ccmFc, ccmFn, cob, cox1, cox2, cox3, nad1, nad2, nad3, nad4, nad4L, nad5, nad6, nad7 and nad9*) extracted from these 28 plant mt genomes. These nucleotides were aligned using ClustalW and manually modified to eliminate gaps and missing data. Finally, the maximum likelihood (ML) method was used to construct original phylogenetic trees by MEGA 6.0^64^. The bootstrap replications were performed with 1000 according to Felsenstein^65^. The evolutionary distances were computed using the Kimura 2-parameter method^66^ and the tree was rooted with *Marchantia paleacea*. The NCBI taxonomy common tree was described by Federhen^67^ and constructed using the online NCBI taxonomy database (https://www.ncbi.nlm.nih.gov/Taxonomy/CommonTree/wwwcmt.cgi).

## Electronic supplementary material

supplementary information
