# Supplementary material for: Complete sequence of kenaf (Hibiscus cannabinus) mitochondrial genome and comparative analysis with the mitochondrial genomes of other plants
Source: Sci Rep. 2018 Aug 24;8:12714. doi: 10.1038/s41598-018-30297-w (PMC6109132; doi:10.1038/s41598-018-30297-w)
Supplement: Supplementary file 1 — supplementary information [file 41598_2018_30297_MOESM1_ESM.docx]

**Complete sequence of kenaf (*Hibiscus cannabinus*) mitochondrial genome and comparative analysis with the mitochondrial genomes of other plants**

**Xiaofang Liao^1,2,3^, Yanhong Zhao^3^, Xiangjun Kong^2^, Aziz Khan^2^, Bujin Zhou^2^, Dongmei Liu^4^, Muhammad Haneef Kashif^2^, Peng Chen^2^, Hong Wang^5^ & Ruiyang Zhou^2*^**

1 College of Life Sciences and Technology, Guangxi University, Nanning, 530005, China

2 Key Laboratory of Plant Genetic and Breeding, College of Agriculture, Guangxi University, Nanning 530005, China

3 Cash Crop Institute of Guangxi Academy of Agricultural Sciences, Nanning, 530007, China

4 Key Laboratory of Plant-Microbe Interactions, Department of Life Science and Food, Shangqiu Normal University, Shangqiu, 476000, China

5 Department of Biochemistry, University of Saskatchewan, Saskatoon, SK, S7N 5E5, Canada

*Correspondence: ruiyangzhou@aliyun.com;


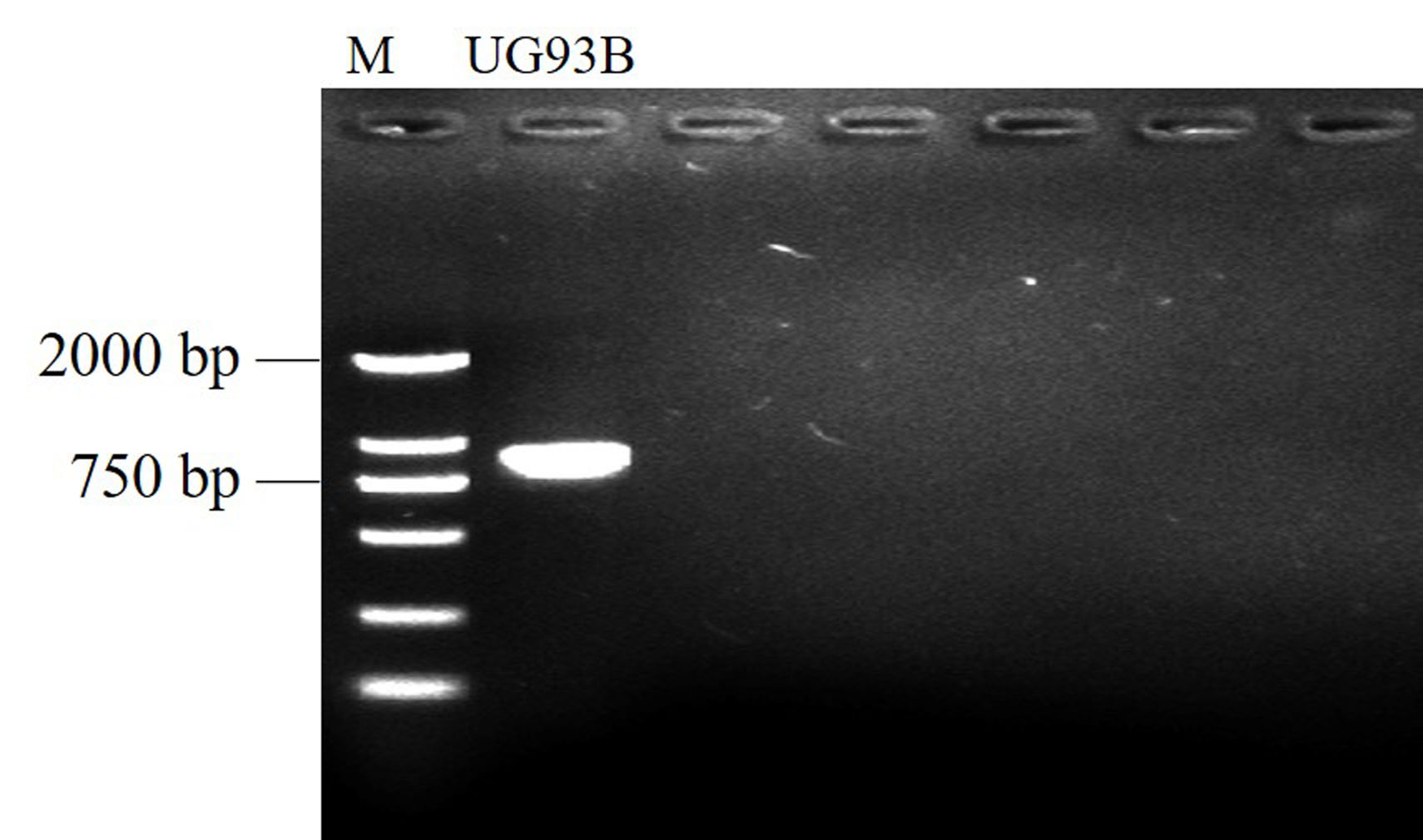


**Fig. S1.** Circular amplification of kenaf mitochondrial.

Forward primer (F: ATGAGAATCCGTACAAGCAAAGACA) was designed at the position of 569,624 bp of kenaf mitochondrial genome, reverse primer (R: GATTGGGATTATAGGTTTCGGCT) was designed at the position of 587 bp. Total production was 878 bp. PCR product was directly sequenced by ABI3730XL sequencer.


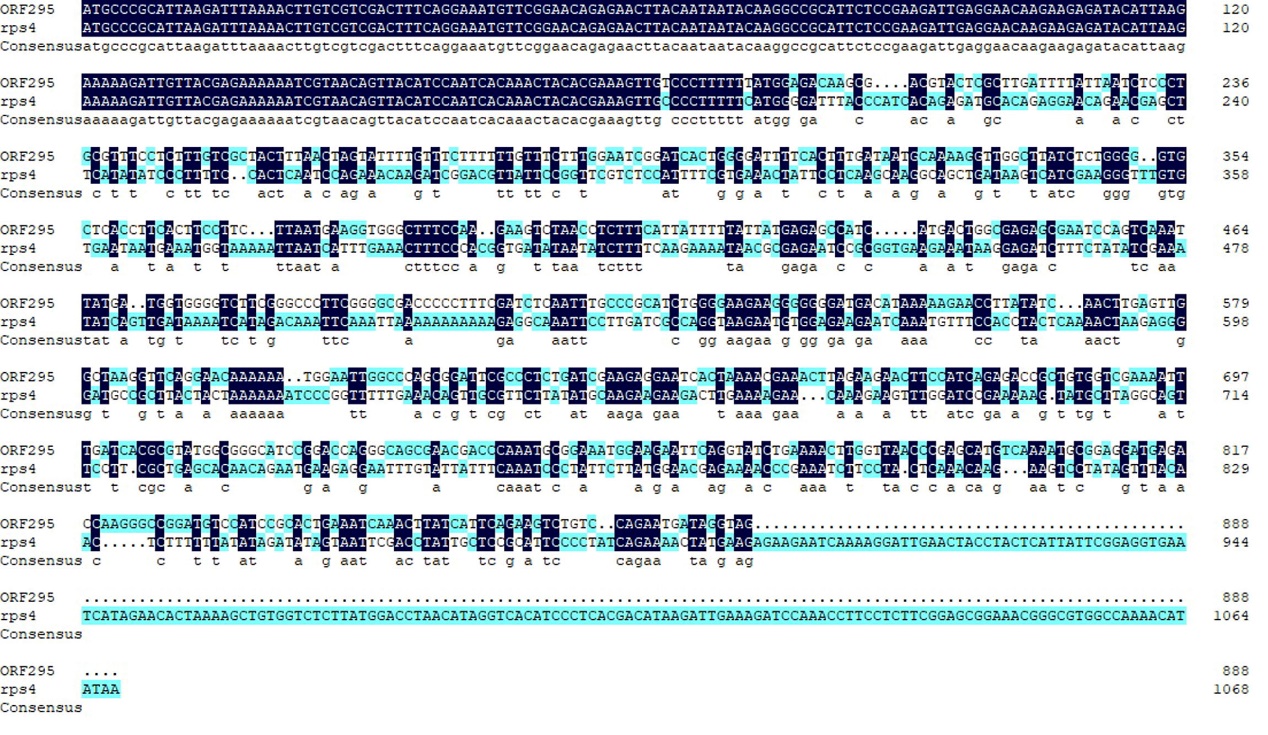


**Fig. S2.** The alignment of the *ORF295* and *rps4* genes in the kenaf mitochondrial genome.

**Fig. S3.** Distribution of repeats in kenaf mitochondrial genome.


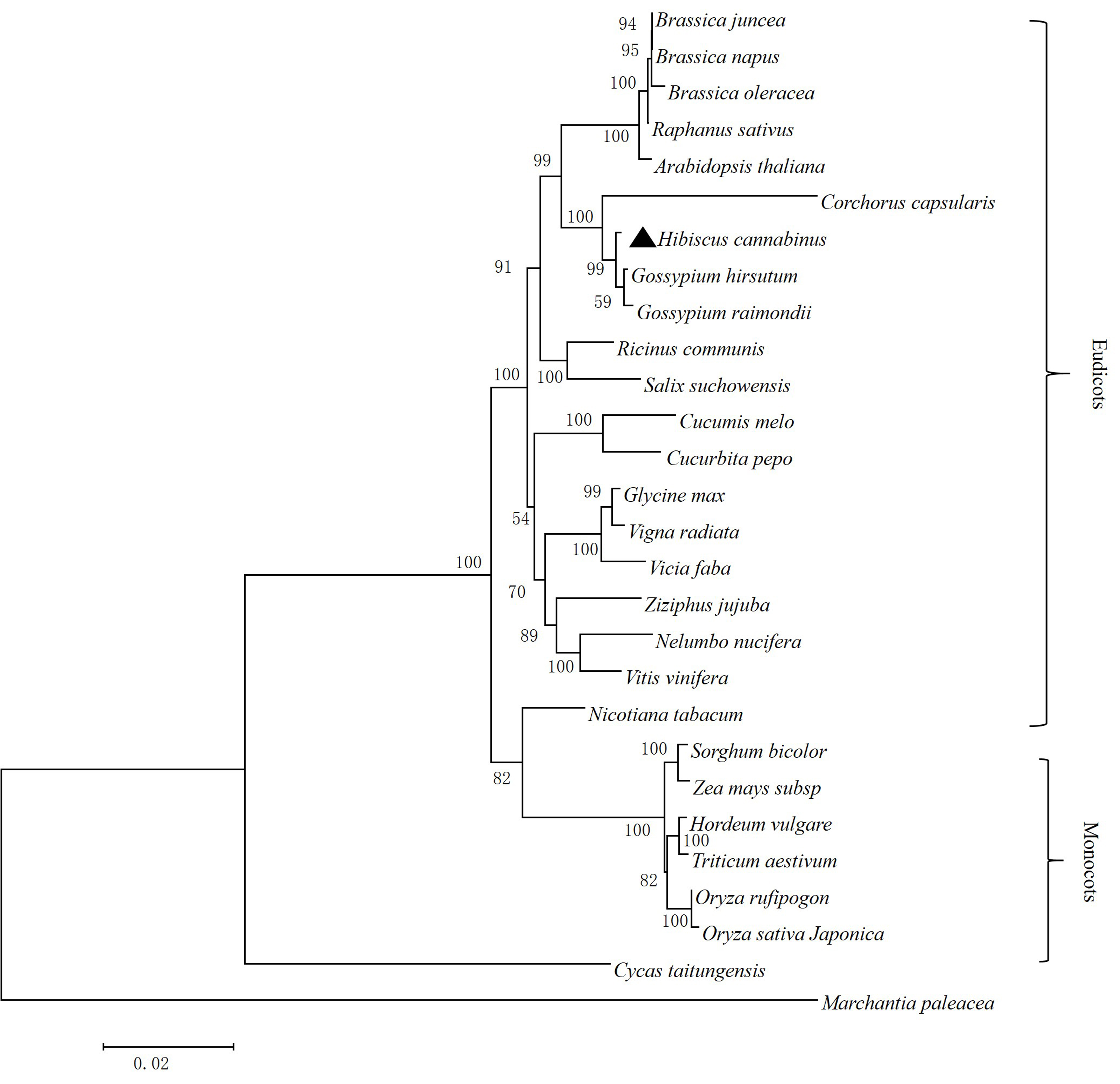


**Fig. S4.** Phylogenetic tree of kenaf mitochondrial complex I.


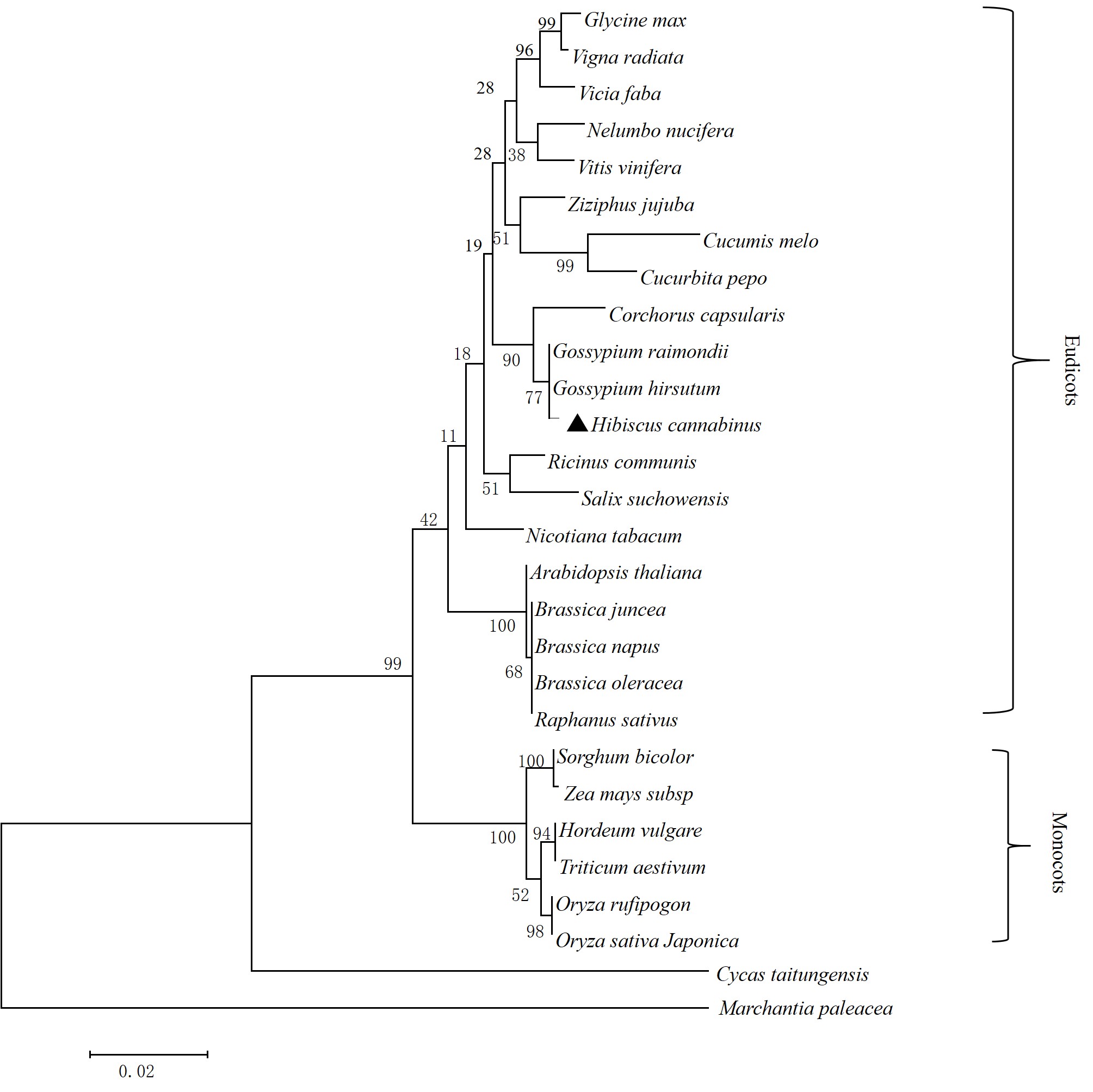


**Fig. S5.** Phylogenetic tree of kenaf mitochondrial complex III*.*


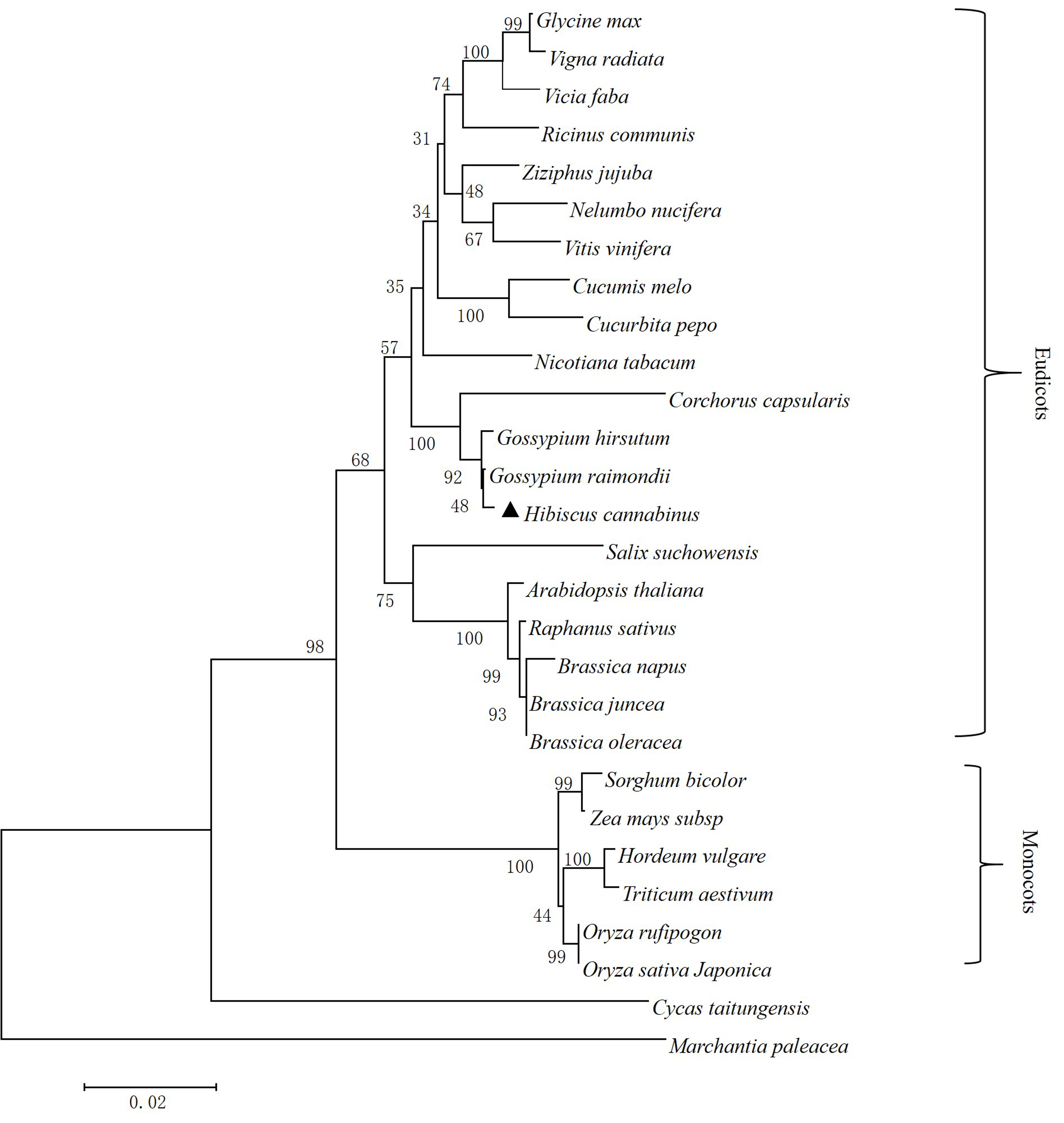


**Fig. S6.** Phylogenetic tree of kenaf mitochondrial complex IV.


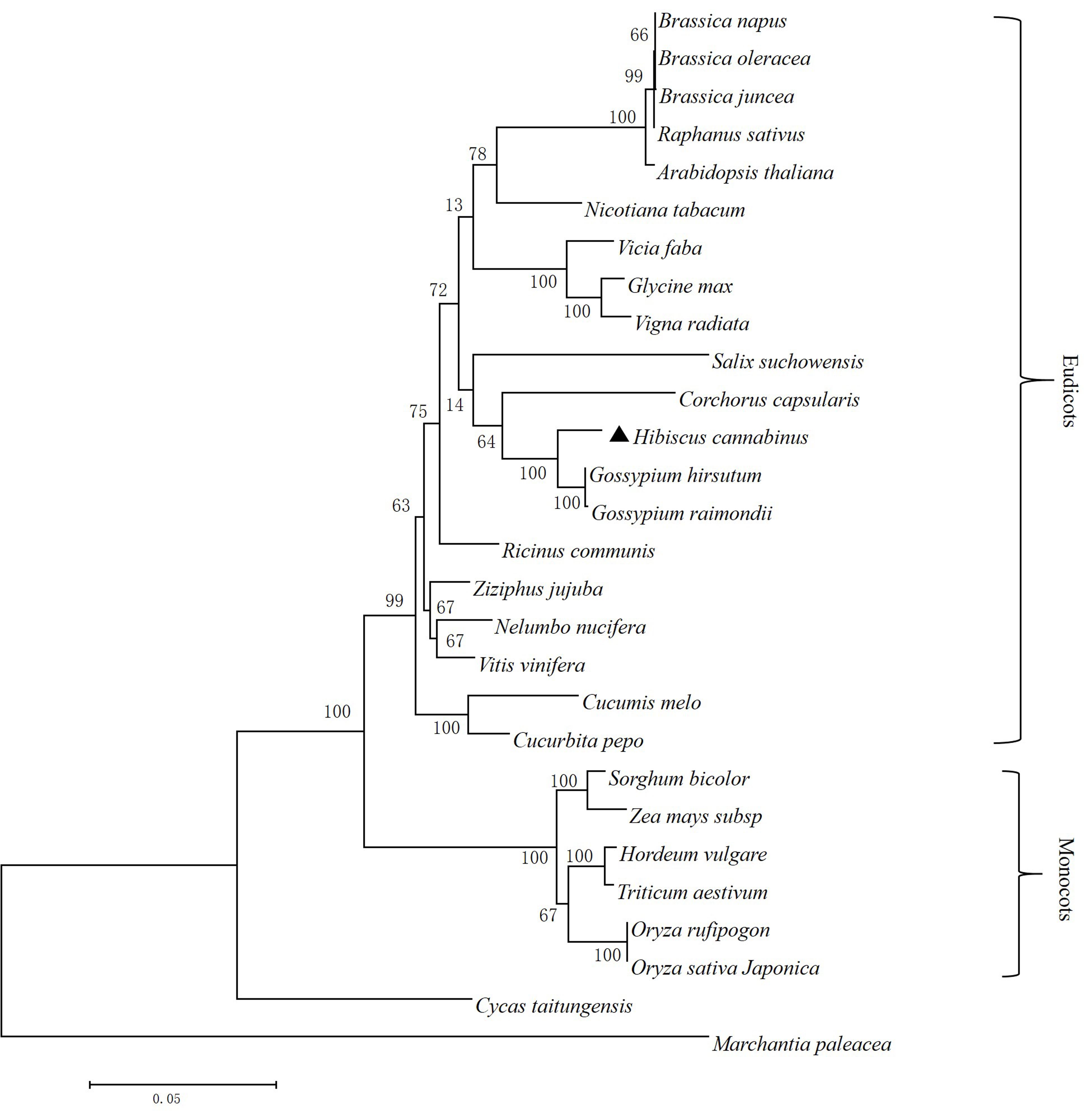


**Fig. S7.** Phylogenetic tree of kenaf mitochondrial complex V.


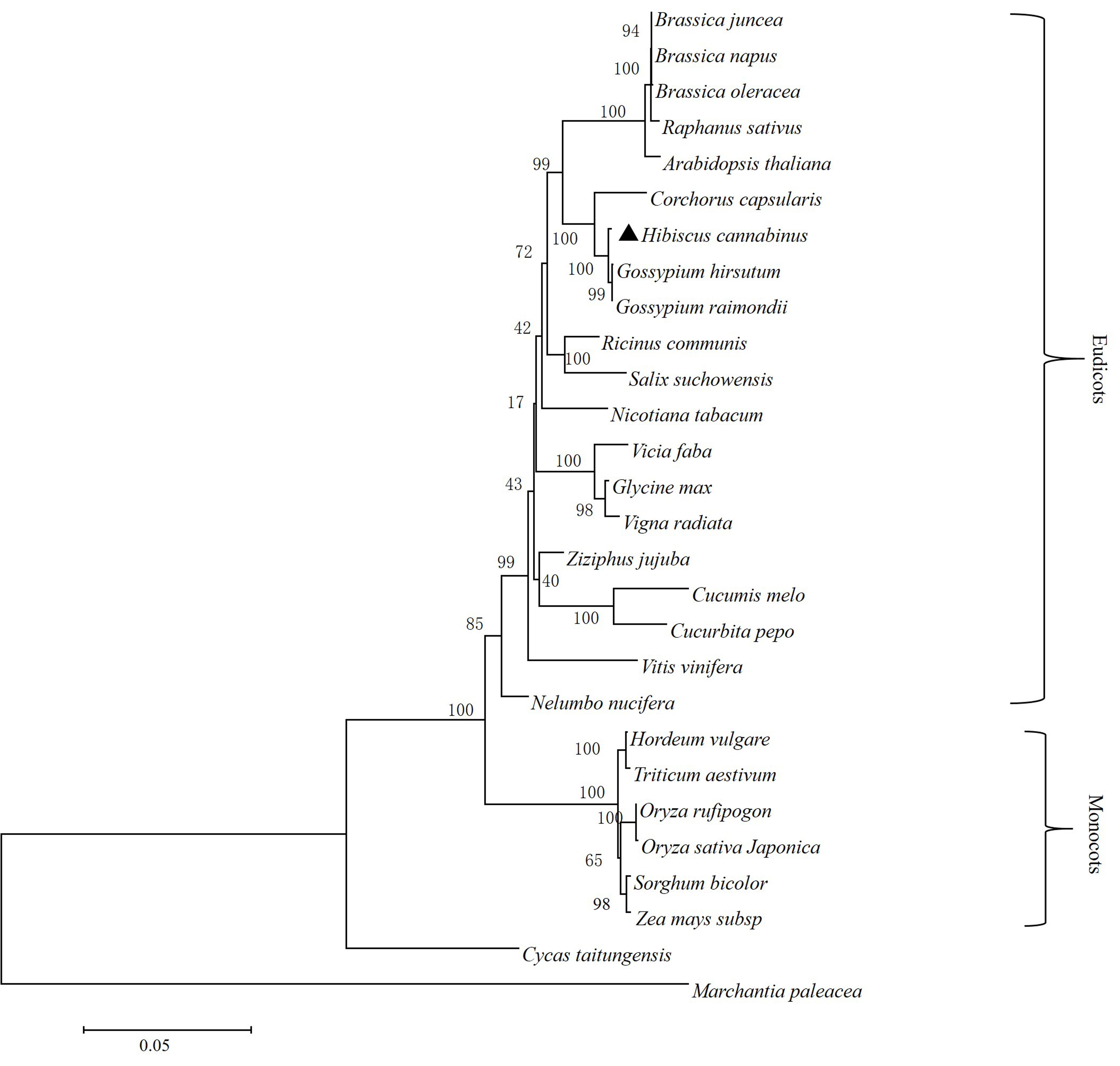


**Fig. S8.** Phylogenetic tree of mitochondrial gene cytochrome c biogenesis genes.

**Table S1.** Sequencing data statistics for kenaf mtDNA from PacBio RSII.

| Sample | Post-Filter No. of Bases | Post-Filter No. of Reads | Post-Filter Mean Read Length | Post-Filter  Mean Read Quality | No. of SubReads | Mean Subread Length | Longest read |
| --- | --- | --- | --- | --- | --- | --- | --- |
| UG93B | 363,717,023 | 67,152 | 5,416 | 0.81 | 78,861 | 4,604 | 3,2332 |

**Table S2.** Composition of the kenaf mt genome.

| Category | Feature | Number | bp (%) |
| --- | --- | --- | --- |
| Genome | G+C |  | 255,961 (44.9%) |
| Genes (total) | | | |
|  | Protein coding | 36 | 39,534 (6.9%) |
|  | rRNA | 3 | 5,265 (0.9%) |
|  | mt-derived tRNA | 21 | 1,590 (0.2%) |
|  | cp-derived tRNA | 8 | 623 (0.1%) |
|  | ORF | 126 | 54,750 (9.6%) |
| Introns | | | |
|  | *cis*-spliced | 20 | 20,301 (3.6%) |
|  | *trans-*spliced | 5 | not know |
| Repeats | | | |
|  | Larger repeats (> 1000) | 3 | 22,368 (3.92%) |
|  | Medium repeats (> 100 , < 999) | 25 | 12,970 (2.28%) |
|  | Short repeats (> 20, < 99) | 556 | 31,375 (5.50%) |

**Table S3.** Prediction and annotation of mtDNA in kenaf.

| Gene types | Genes | Numbers | |
| --- | --- | --- | --- |
| ATP synthase | *atp1, atp4, atp6, atp8, atp9*(2)^*^ | | 5 |
| Cytochrome c oxidase | *cox1, cox2^#^, cox3* | | 3 |
| Cytochrome c biogenesis | *ccmB, ccmC, ccmFc^#^, ccmFn* | | 4 |
| NADH dehydrogenase | *nad1^#^, nad2^#^, nad3, nad4^#^, nad4L, nad5^#^, nad6, nad7^#^, nad9* | | 9 |
| Ribosomal protein | *rpl2, rpl5, rpl10, rpl16, rps3^#^, rps4, rps7, rps10^#^, rps12, rps14, rps19,* | | 11 |
| Ribosomal RNA | *rrn5, rrnL, rrnS* | | 3 |
| Transfer RNA | *trnD, trnG,* trnM(3)^*^, *trnS*(2)^*^*, trnH-cp, trnF, trnP, trnE, trnC, trnN, trnY,* trnP-cp, *trnW-cp, trnfM*(3)^*^*,trnD-cp, trnK, trnQ*(2)^*^*, trnS-cp,* t*rnM-cp, trnL, trnI, trnI-cp, trnV-cp,* | | 23 |
| Others | *sdh4, matR, mttB, cob* | | 4 |
| Total |  | | 62 |

The numbers in parentheses indicate the copy number of the gene. Genes with introns are presented with a pound sign (#).

**Table S4.** Annotated genes in the kenaf mt genome

| Type | Gene | Location |  | Type | Gene | Location |
| --- | --- | --- | --- | --- | --- | --- |
| Complex I | *nad1*^a,b^ | 20583…20967 |  | Ribosomal proteins (LSU) | *rpl2* | **49052…50056** |
|  |  | 359636…359718 |  |  | *rpl5* | **50552…51133** |
|  |  | 360608…360799 |  |  | *rpl10* | 92188…92676 |
|  |  | 535671…535729 |  |  | *rpl16* | **44912…45469** |
|  |  | 539715…539414 |  | Ribosomal proteins (SSU) | *rps3* ^a^ | **45318…45970** |
|  | *nad2*^a,b^ | 205895…206047 |  |  |  | **46016…46634** |
|  |  | 207259…207650 |  |  |  | **46676 …46921** |
|  |  | 319386…319546 |  |  |  | **48595…48696** |
|  |  | 322009…322581 |  |  | *rps4* | 84655…85722 |
|  |  | 323926…324113 |  |  | *rps7* | 152794…153240 |
|  | *nad3* | 284435…284791 |  |  | *rps10*^a^ | 191110…191359 |
|  | *nad4*^a^ | 336894…337352 |  |  |  | 192209…192321 |
|  |  | 338767…339283 |  |  | *rps12* | 227176…227547 |
|  |  | 342198…342619 |  |  | *rps14* | **271693…271965** |
|  |  | 345369…345458 |  |  | *rps19* | **130347…130538** |
|  | *nad4L* | 495757…496059 |  | Other genes | *matR* | 536391…538358 |
|  | *nad5* ^a,b^ | **268246…268481** |  |  | *mttB* | 249953…250753 |
|  |  | **266193…267396** |  | Transfer RNAs | *trnI-cp* | **10535…10620** |
|  |  | 414962…415357 |  |  | *trnG* | 34473…34544 |
|  |  | 416289…416438 |  |  | *trnfM-1* | 63055…62989 |
|  | *nad6* | **492156…492776** |  |  | *trnfM-2* | **136844…136910** |
|  | *nad7^a^* | 234822…234982 |  |  | *trnfM-3* | 358471…358537 |
|  |  | 237487…237953 |  |  | *trnI* | 91349…91263 |
|  |  | 239016…239259 |  |  | *trnM-1* | 118913…118986 |
|  |  | 241069…241330 |  |  | *trnM-2* | 287956…287884 |
|  | *nad9* | 349317…349889 |  |  | *trnM-3* | 413672…413744 |
| Complex II | *sdh4* | **134477…134869** |  |  | *trnS-1* | 120602…120688 |
| Complex III | *cob* | **59475…60653** |  |  | *trnS-2* | 174104…174191 |
| Complex IV | *cox1* | 192476…194077 |  |  | *trnV-cp* | **121998…122069** |
|  | *cox2 ^a^* | 512002…512698 |  |  | *trnH-cp* | 147965…148038 |
|  |  | 514190…514275 |  |  | *trnF* | 174543…174616 |
|  | *cox3* | **134797…135639** |  |  | *trnP* | 174836…174910 |
| Complex V | *atp1* | **269158…270681** |  |  | *trnL* | 219515…219604 |
|  | *atp4* | 539630…540196 |  |  | *trnQ-1* | **256866…256937** |
|  | *atp6* | **470103…471296** |  |  | *trnQ-2* | 501903…501974 |
|  | *atp8* | 91579…92058 |  |  | *trnM-cp* | **274364…274436** |
|  | ***atp9-1*** | **4544…4807** |  |  | *trnE* | 289694…289765 |
|  | *atp9-2* | 200418…200681 |  |  | *trnC* | 315661…315731 |
| Cytochrome c biogenesis | *ccmB* | **442729…443349** |  |  | *trnN* | 317829…317900 |
|  | *ccmC* | **10607…11359** |  |  | *trnY* | 318786…318868 |
|  | *ccmFc*^a^ | 113150…113922 |  |  | *trnD* | **333467…333540** |
|  |  | 114871…115450 |  |  | *trnP-cp* | 350633…350706 |
|  | *ccmFn* | **222025…223761** |  |  | *trnW-cp* | 350855…350928 |
| Ribosomal  RNAs | *rrn5* | **399374…399492** |  |  | *trnD-cp* | 429242…429315 |
|  | *rrnL* | **377473…380665** |  |  | *trnK* | 445685…445757 |
|  | *rrnS* | **399656…401605** |  |  | *trnS-cp* | **542340…542426** |

a: genes contain introns; b: genes that need *trans*-splicing; boldface: transcription occurs on the antisense strand.

**Table S5.** Distribution of chloroplast sequences in the kenaf mitochondrial genome.

| Sequence | Position | Gene |
| --- | --- | --- |
| Cp-1 | **10537-10621** | ***trnI-cp*** |
| Cp-2 | 66693-67213 |  |
| Cp-3 | **121533-124185** | *t****rnV-cp*** |
| Cp-4 | 147964-148029 | ***trnH-cp*** |
| Cp-5 | 159532-159764 |  |
| Cp-6 | **274364-274436** | ***trn M-cp*** |
| Cp-7 | **324940-328603** | **partial *psaB, psaA*** |
| Cp-8 | 350529-351035 | *trnP-cp，trnW-cp* |
| Cp-9 | 429242-429431 | *trnD-cp* |
| Cp-10 | 473828-474459 | partial *psbD* |
| Cp-11 | **542283-542679** | ***trnS-cp Ser*** |
| Cp-12 | **566988-569474** | ***ndhB, rps7*** |

boldface: transcription occurs on the antisense strand.

**Table S6.** Distribution of gene clusters in land plant mitochondrial genomes.

|  | *rrn18-rrn5* | *nad5-nad1-matR* | *rps12-nad3* | *(rps19)-rps3-rpl16* | *atp4-nad4L* | *rps10-cox1* | *sdh4-cox3-(atp8)* | *rpl5-rps14* | *rpl2-rpl5* |
| --- | --- | --- | --- | --- | --- | --- | --- | --- | --- |
| *M. paleacea* | **+** | **+** | **-** | **+** | **-** | **-** | **-** | **+** | **-** |
| *C. taitungensis* | **+** | **+** | **+** | **+** | **-** | **+** | **-** | **+** | **-** |
| *H. vulgare* | **+** | **+** | **+** | **+** | **-** | **/** | **-** | **/** | **/** |
| *O. rufipogon* | **+** | **+** | **+** | **+** | **-** | **-** | **-** | **+** | **-** |
| *O. sativa* | **+** | **+** | **+** | **+** | **-** | **-** | **-** | **-** | **-** |
| *S. bicolor* | **+** | **+** | **+** | **+** | **-** | **-** | **-** | **-** | **-** |
| *T. aestivum* | **+** | **+** | **+** | **+** | **-** | **-** | **-** | **/** | **/** |
| *Z. mays subsp* | **+** | **+** | **+** | **+** | **-** | **-** | **/** | **/** | **/** |
| *A. thaliana* | **+** | **+** | **-** | **+** | **-** | **-** | **-** | **/** | **/** |
| *B. juncea* | **+** | **+** | **+** | **+** | **+** | **-** | **-** | **+** | **-** |
| *B. napus* | **+** | **+** | **+** | **+** | **+** | **-** | **-** | **+** | **-** |
| *B. oleracea* | **+** | **+** | **+** | **+** | **+** | **-** | **-** | **+** | **-** |
| *C. capsularis* | **+** | **+** | **+** | **+** | **-** | **+** | **+** | **-** | **-** |
| *C.* *lanatus* | **+** | **+** | **+** | **+** | **+** | **+** | **+** | **+** | **-** |
| *C. melo* | **+** | **+** | **+** | **+** | **-** | **+** | **+** | **/** | **/** |
| *C. pepo* | **+** | **+** | **+** | **+** | **+** | **+** | **+** | **+** | **-** |
| *H. cannabinus* | **+** | **+** | **+** | **+** | **-** | **+** | **+** | **-** | **-** |
| *G. max* | **+** | **+** | **+** | **+** | **+** | **+** | **/** | **/** | **/** |
| *G. raimondii* | **+** | **+** | **+** | **+** | **-** | **+** | **+** | **-** | **+** |
| *G. hirsutum* | **+** | **+** | **+** | **+** | **-** | **+** | **+** | **-** | **+** |
| *N. nucifera* | **+** | **+** | **+** | **+** | **+** | **+** | **+** | **+** | **-** |
| *N. tabacum* | **+** | **+** | **+** | **+** | **-** | **+** | **-** | **+** | **-** |
| *R. communis* | **+** | **+** | **+** | **+** | **+** | **+** | **+** | **-** | **-** |
| *R. sativus* | **+** | **+** | **+** | **+** | **+** | **-** | **-** | **+** | **-** |
| *S. suchowensis* | **+** | **+** | **-** | **-** | **+** | **-** | **+** | **/** | **/** |
| *V. faba* | **+** | **+** | **+** | **+** | **+** | **+** | **/** | **+** | **-** |
| *V. radiata* | **+** | **+** | **+** | **+** | **+** | **+** | **+** | **+** | **-** |
| *V. vinifera* | **+** | **+** | **+** | **+** | **+** | **-** | **+** | **+** | **-** |
| *Z. jujuba* | **+** | **+** | **+** | **+** | **+** | **+** | **+** | **+** | **-** |

**+**, indicates that the cluster exists in plant mtDNA; **-**, indicates that the cluster is absent in plant mtDNA; **/**, indicates that the cluster is lost.
